# Association of Intellectual Disability With All-Cause and Cause-Specific Mortality in Sweden

**DOI:** 10.1001/jamanetworkopen.2021.13014

**Published:** 2021-06-22

**Authors:** Tatja Hirvikoski, Marcus Boman, Magnus Tideman, Paul Lichtenstein, Agnieszka Butwicka

**Affiliations:** 1 Department of Women’s and Children’s Health, Pediatric Neuropsychiatry Unit, Center for Neurodevelopmental Disorders at Karolinska Institutet, Karolinska Institutet, Stockholm, Sweden; 2 Habilitation and Health, Stockholm Health Care Services, Region Stockholm, Stockholm, Sweden; 3 Center for Psychiatry Research, Stockholm County Council, Stockholm, Sweden; 4 Department of Medical Epidemiology and Biostatistics, Karolinska Institutet, Stockholm, Sweden; 5 School of Health and Welfare, Halmstad University, Halmstad, Sweden; 6 Child and Adolescent Psychiatry Stockholm, Stockholm Health Care Services, Region Stockholm, Stockholm, Sweden; 7 Department of Child Psychiatry, Medical University of Warsaw, Warsaw, Poland

## Abstract

**Importance:**

Knowledge of the health challenges and mortality in people with intellectual disability (ID) should guide health policies and practices in contemporary society.

**Objective:**

To examine premature mortality in individuals with ID.

**Design, Setting, and Participants:**

This population-based longitudinal cohort study obtained data from several national health care, education, and population registers in Sweden. Two registers were used to identify individuals with ID: the National Patient Register and the Halmstad University Register on Pupils With Intellectual Disability. Two cohorts were created: cohort 1 comprised young adults (born between 1980 and 1991) with mild ID, and cohort 2 comprised individuals (born between 1932 and 2013) with mild ID or moderate to profound ID; each cohort had matched reference cohorts. Data analyses were conducted between June 1, 2020, and March 31, 2021.

**Exposures:**

Mild or moderate to profound ID.

**Main Outcomes and Measures:**

The primary outcome was overall (all-cause) mortality, and the secondary outcomes were cause-specific mortality and potentially avoidable mortality.

**Results:**

Cohort 1 included 13 541 young adults with mild ID (mean [SD] age at death, 24.53 [3.66] years; 7826 men [57.8%]), and its matched reference cohort consisted of 135 410 individuals. Cohort 2 included 24 059 individuals with mild ID (mean [SD] age at death, 52.01 [16.88] years; 13 649 male individuals [56.7%]) and 26 602 individuals with moderate to profound ID (mean [SD] age at death, 42.16 [21.68] years; 15 338 male individuals [57.7%]); its matched reference cohorts consisted of 240 590 individuals with mild ID and 266 020 with moderate to profound ID. Young adults with mild ID had increased overall mortality risk compared with the matched reference cohort (odds ratio [OR], 2.86; 95% CI, 2.33-3.50), specifically excess mortality in neoplasms (OR, 3.58; 95% CI, 2.02-6.35), diseases of the nervous system (OR, 40.00; 95% CI, 18.43-86.80) and circulatory system (OR, 9.24; 95% CI, 4.76-17.95). Among deaths that were amenable to health care (OR, 7.75; 95% CI, 4.85-12.39), 55% were attributed to epilepsy. In cohort 2, increased risk of overall mortality was observed among both individuals with mild ID (OR, 6.21; 95% CI, 5.79-6.66) and moderate to profound ID (OR, 13.15; 95% CI, 12.52-13.81) compared with the matched reference cohorts. Those with moderate to profound ID had a higher risk in several cause-of-death categories compared with those with mild ID or the matched reference cohort. Adjustment for epilepsy and congenital malformations attenuated the associations. The relative risk of premature death was higher in women (OR, 6.23; 95% CI, 4.42-8.79) than in men (OR, 1.99; 95% CI, 1.53-2.60), but the absolute risk of mortality was similar (0.9% for women vs 0.9% for men).

**Conclusions and Relevance:**

This study found excess premature mortality and high risk of deaths with causes that were potentially amenable to health care intervention among people with ID. This finding suggests that this patient population faces persistent health challenges and inequality in health care encounters.

## Key Points

**Question:**

What health challenges as indicated by premature mortality do people with intellectual disability (ID) face?

**Findings:**

In this population-based cohort study of 13 541 young adults with mild ID, 24 059 individuals with mild ID, 26 602 individuals with moderate to profound ID, and hundreds of thousands of control individuals in Sweden, premature mortality was significantly increased in those with ID. Excess risk of mortality observed in young adults with mild ID may be attributed to several specific causes of death and potentially treatable diagnoses.

**Meaning:**

Results of this study suggest that people with ID grapple with persistent health challenges and a high burden of disease that is associated with premature mortality in a contemporary welfare society.

## Teaser

This population-based cohort study evaluates the risks and prevalence of premature mortality in individuals with varying severity of intellectual disability in Sweden.

## Introduction

Intellectual disability (ID) is a neurodevelopmental disorder characterized by substantial limitations on both intellectual ability and adaptive functioning; in most cases, these limitations lead to lifelong impairments and the need for support in many major life activities.^1^ However, the nature and extent of these impairments vary greatly depending on the severity of the ID. Compared with the general population, those with ID have poor physical and mental health.^2,3,4^ In addition, ID has been associated with shorter life expectancy of up to 2 decades and low mean age at death,^5,6^ although the mortality discrepancy between those with ID and the general population has decreased in the past decades.^5^ The increase in mean age at death in ID may illustrate the improvements in policies and practices, including the deinstitutionalization initiated in many high-income countries during the 1970s and 1980s that eventually led to the complete closure of large institutions in Sweden.^7^ Living in the community instead of in congregate arrangements is generally associated with improved quality of life and several other outcomes,^8^ but some studies have also indicated that limitations persist in accessing health care in community settings.^6,8^ Therefore, studies into the health experiences of people with ID after deinstitutionalization are important to understanding the health challenges for people with ID in a contemporary welfare society.

The discrepancies in overall mortality risk in previous studies^5,6,9,10,11,12,13,14,15^ may be explained by methodological differences, such as sample selection, inclusion of narrow age ranges, and lack of matched control populations. Therefore, nationwide population-based studies with control individuals who are matched to individuals with different severity levels of ID are needed to increase the generalizability of the results. Furthermore, specific causes of death in mild ID compared with more severe ID (moderate to profound) have been identified as a knowledge gap in existing literature,^14^ and studies of the factors associated with excess mortality^16,17,18,19,20,21,22^ at different severity levels of ID are scarce. Moreover, some studies indicated that the overall mortality risk was generally higher among female individuals with ID,^5,6,9,11,14^ but this observation was not confirmed in other studies.^13,15^ The current scarcity of high-quality, population-based studies on mortality in individuals with ID may be inherent in the poor coverage of registries; for example, the Swedish National Patient Register (NPR) captures only a select minority of people with ID.

To examine premature mortality in individuals with ID, we performed a population-based study using nationwide health care and education registers in Sweden. We estimated the risk of premature all-cause, cause-specific, and potentially avoidable mortality, focusing on young adults with mild ID living in community settings.

## Methods

This cohort study was approved by the Regional Ethical Review Board in Stockholm, Sweden. No patient consent was obtained because the work was a register-based epidemiological study. We followed the Strengthening the Reporting of Observational Studies in Epidemiology (STROBE) reporting guideline.

### Study Design and Setting

To conduct a population-based cohort study, we linked several nationwide population-based registers using the unique personal identification number that all Swedish residents have. Two registers were used to identify individuals with ID: the NPR and Halmstad University Register on Pupils With Intellectual Disability (HURPID).

The NPR applies diagnostic codes that are based on the Swedish versions of the *International Classification of Diseases, Eighth Revision* (*ICD-8*); *International Classification of Diseases, Ninth Revision* (*ICD-9*); and *International Statistical Classification of Diseases and Related Health Problems, Tenth Revision* (*ICD-10*). The NPR includes primary and secondary psychiatric diagnoses for inpatient care (from 1973 onward) and outpatient care (from 2001 onward), including a diagnostic assessment for ID without any further contact with psychiatric services. The HURPID is a national database of persons (n = 12 269) who graduated from an upper secondary school for students with intellectual disability (USSID) between January 1, 2001, and December 31, 2011.^23,24^ Persons in the HURPID database are coded according to the USSID program they attended,^23^ such as national programs (eg, special programs that focus on sports) and individual programs (eg, those that are adjusted for individual needs, such as vocational training and activity training).^25^ Assignment to these USSID programs is based on the student’s profile, which is created from psychological, pedagogical, medical, and social assessments.^26^ Because activity training programs are adjusted for students with greater needs, most of the students who attend these programs have moderate to profound ID.

### Study Cohorts

We created 2 cohorts: cohort 1 comprised young adults with mild ID, and cohort 2 comprised individuals with mild or moderate to profound ID. All individuals were followed up from their inclusion in the study until the end of the study period (December 31, 2013) or death, whichever occurred first.

To focus on the young adults with mild ID in the contemporary Swedish welfare society, we restricted cohort 1 to include individuals who were born between January 1, 1980, to December 31, 1991; alive at 18 years of age; and identified either from the NPR or the HURPID database (eFigure 1 in the Supplement). The categorization of ID from the NPR and the HURPID databases is depicted in eTable 1 in the Supplement, whereas the correspondence of the 2 registers is depicted in eTable 2 in the Supplement.

To enable a comparison to previous studies on mortality in ID, including all levels of ID severity and enrollment over a long period, we examined mortality in a second cohort from the NPR. In cohort 2, we included all individuals with ID diagnoses identified from 1969 to 2013 who were born between January 1, 1932, and December 31, 2013 (eFigure 1 in the Supplement). The categorization to mild vs moderate to profound ID is described in eTable 1 in the Supplement.

For each individual with ID, we identified 10 reference individuals who were matched from the Total Population Register^27^ (135 410 for cohort 1, and 506 610 for cohort 2). The reference individuals were required to be free from ID; to be the same sex; to be born during the same calendar year; and to be alive and living in the same county in Sweden as the individuals with ID when they were first diagnosed, as recorded in the NPR, or when they finished the USSID (exact matching).

### Outcomes and Confounding Factors

The primary outcome of the study was overall (all-cause) mortality, and the secondary outcomes were cause-specific mortality, categorized by the chapters in the *ICD*,^28^ and potentially avoidable mortality^29^ (defined in eTable 3 in the Supplement). The *ICD* diagnostic codes were extracted from the Cause of Death Register.

To analyze the role of possible confounding factors (described in eTable 1 in the Supplement), we conducted a series of analyses adjusted for (1) parental educational level using 3 data sources from Statistics Sweden; (2) congenital malformations, deformations, and chromosomal abnormalities; (3) epilepsy; (4) other coexisting neurodevelopmental disorders, such as autism spectrum disorder and/or attention-deficit/hyperactivity disorder; and (5) psychiatric comorbidity, such as depression and/or anxiety disorders.

### Statistical Analysis

Conditional logistic regression analyses were conducted to calculate odds ratios (ORs) with 95% CIs for all-cause, cause-specific, and potentially avoidable mortality. Analyses for all-cause mortality were stratified by sex in cohort 1 and sex and level of ID in cohort 2. In addition to the crude OR analysis, we conducted a series of adjusted analyses to examine the potential role of possible confounding factors in separate models. Kaplan-Meier survival plots were drawn for the 2 cohorts and their matched reference cohorts and are shown in eFigure 2 in the Supplement.

All analyses were planned a priori. Data are not shown for any cell that included fewer than 5 individuals. We conducted the statistical analyses between June 1, 2020, and March 31, 2021, using SAS, version 9.4 (SAS Institute Inc).

## Results

### Cohort 1: Young Adults With Mild ID

A total of 13 541 young adults with mild ID were included in cohort 1, whereas 135 410 individuals composed the matched reference cohort. The young adults in cohort 1 had a mean (SD) age at death of 24.53 (3.66) years and were composed of 7826 men (57.8%) and 5715 women (42.2%). The demographic data of cohort 1 are depicted in Table 1.

**Table 1.** Demographic Characteristics of Cohort 1: Young Adults With Mild Intellectual Disability (ID) and Their Matched Reference Cohort

Characteristic	No. (%)	
	Cohort 1: with mild ID	Matched reference cohort
Total No. of individuals	13 541	135 410
Sex		
Male	7826 (57.8)	78 260 (57.8)
Female	5715 (42.2)	57 150 (42.2)
Age at first ID diagnosis in the NPR, mean (SD) [range], y	17.97 (5.82) [0.06-33.60]	NA
Age at finishing the USSID, mean (SD) [range], y	20.60 (0.74) [18.04-23.96]	NA
No. of deaths	120 (0.9)	424 (0.3)
Age at death, y		
All individuals		
Mean (SD)	24.53 (3.66)	24.57 (3.00)
Median (range)	24.22 (18.11-32.76)	24.17 (18.10-32.71)
Male individuals		
Mean (SD)	25.24 (3.69)	24.41 (2.97)
Median (range)	25.36 (18.69-32.76)	24.05 (18.10-32.71)
Female individuals		
Mean (SD)	23.64 (3.47)	25.19 (3.05)
Median (range)	23.57 (18.11-32.21)	25.11 (18.61-32.14)
Parental educational level		
High: academic	2941 (21.7)	60 103 (44.4)
Middle: upper secondary	7912 (58.4)	62 690 (46.3)
Low: elementary, ≤9 y compulsory	1975 (14.6)	8185 (6.0)
Missing data	713 (5.3)	4432 (3.3)
Coexisting diagnoses		
*ICD-10* Chapter XVII: Chromosomal	2887 (21.3)	8373 (6.2)
Epilepsy	1453 (10.7)	1355 (1.0)
ASD and/or ADHD	3492 (25.8)	3815 (2.8)
Depression and/or anxiety	2565 (18.9)	11 712 (8.7)

Abbreviations: ADHD, attention-deficit/hyperactivity disorder; ASD, autism spectrum disorder; *ICD-10 *Chapter XVII: Chromosomal*, International Statistical Classification of Diseases and Related Health Problems, Tenth Revision,* Chapter XVII: Congenital malformations, deformations, and chromosomal abnormalities; NA, not applicable; NPR, Swedish National Patient Register; USSID, upper secondary school for students with intellectual disability.

During follow-up, 120 young adults with mild ID (0.9%) died (Table 2; eFigure 2 in the Supplement) compared with 424 individuals (0.3%) in the matched reference cohort. Thus, the relative risk of premature death was 2.86-fold in the entire cohort 1 (OR, 2.86; 95% CI, 2.33-3.50) and was higher in women (OR, 6.23; 95% CI, 4.42-8.79) than in men (OR, 1.99; 95% CI, 1.53-2.60) (Table 2). However, the absolute risk of mortality was similar (0.9% for women [53 deaths of 5715 individuals] vs 0.9% for men [67 deaths of 7826 individuals]). In both sexes, the adjustment for potential confounders had a marginal effect on excess mortality.

**Table 2.** Risk of All-Cause, Cause-Specific, and Potentially Avoidable Mortality in Cohort 1: Young Adults With Mild Intellectual Disability (ID)^a^

Category	No./Total No. (%)		OR (95% CI)						
	Cohort 1: with mild ID	Matched reference cohort	Crude	Parental educational level	Congenital malformations	Epilepsy	ASD and/or ADHD	Depression and/or anxiety	Adjusted for all potentially confounding factors
Overall or all-cause mortality									
All individuals	120/13 541 (0.9)	424/135 410 (0.3)	2.86 (2.33-3.50)	2.77 (2.25-3.42)	2.66 (2.15-3.28)	2.25 (1.80-2.81)	2.44 (1.94-3.06)	2.37 (1.93-2.92)	1.68 (1.31-2.15)
Male individuals	67/7826 (0.9)	338/78 260 (0.4)	1.99 (1.53-2.60)	1.91 (1.46-2.50)	1.90 (1.45-2.49)	1.64 (1.24-2.18)	1.58 (1.17-2.12)	1.63 (1.25-2.13)	1.20 (0.87-1.64)
Female individuals	53/5715 (0.9)	86/57 150 (0.2)	6.23 (4.42-8.79)	6.31 (4.42-9.02)	5.47 (3.82-7.85)	4.58 (3.12-6.72)	5.90 (4.08-8.54)	5.28 (3.71-7.50)	3.53 (2.30-5.42)
Cause-specific mortality by *ICD-10* chapters^b^									
Chapter II: Neoplasms	16/13 541 (0.1)	45/135 410 (0.0)	3.58 (2.02-6.35)	3.26 (1.81-5.88)	3.11 (1.72-5.64)	3.00 (1.59-5.67)	3.63 (1.96-6.70)	3.71 (2.08-6.62)	2.47 (1.21-5.02)
Chapter VI: Nervous system	32/13 541 (0.2)	8/135 410 (0.0)	40.00 (18.43-86.80)	47.15 (21.35-104.13)	37.93 (17.19-83.69)	15.61 (6.57-37.10)	52.26 (23.64-115.56)	37.96 (17.39-82.83)	18.71 (7.62-45.98)
Chapter VI: Nervous system excluding individuals with epilepsy	12/12 088 (0.1)	8/120 880 (0.0)	15.00 (6.13-36.69)	17.25 (6.79-43.83)	13.85 (5.46-35.13)	NA	19.24 (7.60-48.70)	12.67 (5.08-31.63)	19.68 (7.26-53.38)
Chapter IX: Circulatory system	17/13 541 (0.1)	19/135 410 (0.0)	9.24 (4.76-17.95)	9.67 (4.84-19.32)	8.14 (4.06-16.31)	7.45 (3.66-15.13)	10.09 (4.95-20.58)	8.61 (4.38-16.93)	6.64 (2.85-15.49)
Potentially avoidable mortality^c^									
Potentially avoidable, both amenable and preventable	71/13 541 (0.5)	360/135 410 (0.3)	1.98 (1.53-2.56)	1.91 (1.47-2.48)	1.91 (1.47-2.49)	1.57 (1.19-2.07)	1.51 (1.13-2.00)	1.58 (1.22-2.05)	1.09 (0.80-1.48)
Amenable to health care intervention	31/13 542 (0.2)	40/135 410 (0.0)	7.75 (4.85-12.39)	8.55 (5.27-13.89)	6.58 (4.02-10.78)	3.64 (2.10-6.30)	8.82 (5.38-14.44)	7.11 (4.42-11.44)	3.71 (2.08-6.63)
Preventable	44/13 542 (0.3)	333/135 410 (0.3)	1.32 (0.97-1.81)	1.25 (0.91-1.72)	1.32 (0.96-1.82)	1.25 (0.90-1.74)	0.88 (0.62-1.25)	1.03 (0.75-1.41)	0.77 (0.53-1.11)

Abbreviations: ADHD, attention-deficit/hyperactivity disorder; ASD, autism spectrum disorder; *ICD-10, International Statistical Classification of Diseases and Related Health Problems, Tenth Revision*; NA, not applicable; OR, odds ratio.

^a^ Only specific causes of death with significant between-group differences are depicted. The detailed description of the confounders, including diagnostic codes, is provided in eTable 1 in the Supplement.

^b^ Categories with fewer than 5 individuals in any of the cells are not shown.

^c^ Definition is from the Office for National Statistics.^29^

In 3 categories, young adults with mild ID had a significantly higher risk of premature mortality than the matched reference cohort: neoplasms, diseases of the nervous system, and diseases of the circulatory system. The 3 most common causes of death within each category are shown in eTable 4 in the Supplement. In neoplasms (OR, 3.58; 95% CI, 2.02-6.35) and diseases of the circulatory system (OR, 9.24; 95% CI, 4.76-17.95), the risk was generally not associated with the confounders (Table 2). In contrast, in diseases of the nervous system (OR, 40.00; 95% CI, 18.43-86.80), the risk was considerably lower when controlling for epilepsy (adjusted OR, 15.61; 95% CI, 6.57-37.10) or excluding individuals with comorbid epilepsy (Table 2).

The risk of potentially avoidable deaths was 2-fold in young adults with mild ID compared with the matched reference cohort. Although the risk of preventable deaths did not differ significantly between young adults with mild ID and the matched reference cohort, the risk of death with causes that were amenable to health care intervention was almost 8-fold in those with mild ID vs their matched counterparts (OR, 7.75; 95% CI, 4.85-12.39) (Table 2 and Figure). In cohort 1, 55% of amenable mortality was attributed to epilepsy.

### Cohort 2: Individuals With Mild or Moderate to Profound ID

A total of 24 059 individuals with mild ID and 26 602 individuals with moderate to profound ID were included in cohort 2. The matched reference cohorts included 240 590 individuals with mild ID and 266 020 individuals with moderate to profound ID. In cohort 2, those with mild ID had a mean (SD) age at death of 52.01 (16.88) years and included 13 649 male (56.7%) and 10 410 female (43.3%) individuals. Those with moderate to profound ID had a mean (SD) age at death of 42.16 (21.68) years and included 15 338 male (57.7%) and 11 263 female (42.3%) individuals. The demographic data for cohort 2 are depicted in Table 3.

**Table 3.** Demographic Characteristics of Cohort 2: Individuals With Mild or Moderate to Profound Intellectual Disability (ID) and Their Matched Reference Cohorts

Characteristic	No. (%)			
	Cohort 2: with mild ID	Matched reference cohort	Cohort 2: with moderate to profound ID	Matched reference cohort
Total, No. of individuals	24 059	240 590	26 602	266 020
Sex				
Male	13 649 (56.7)	136 490 (56.7)	15 338 (57.7)	153 390 (57.7)
Female	10 410 (43.3)	104 100 (43.3)	11 263 (42.3)	112 630 (42.3)
Age at death, y				
All individuals				
Mean (SD)	52.01 (16.88)	57.76 (15.72)	42.16 (21.68)	57.70 (16.28)
Median (range)	55.08 (0.65-80.53)	61.09 (1.62-81.81)	45.47 (0.34-81.58)	61.65 (0.57-81.88)
Male individuals				
Mean (SD)	50.88 (17.12)	56.74 (16.36)	42.41 (21.48)	56.94 (16.72)
Median (range)	53.80 (0.65-80.34)	60.48 (1.62-81.81)	46.33 (0.55-81.58)	61.15 (1.74-81.88)
Female individuals				
Mean (SD)	53.48 (16.45)	59.41 (14.47)	41.83 (21.94)	59.14 (15.31)
Median (range)	56.62 (3.35-80.53)	62.06 (7.31-81.74)	44.71 (0.34-80.36)	62.57 (0.57-81.79)
Parental educational level				
High: academic	5323 (22.1)	94 550 (39.3)	7214 (27.1)	94 399 (35.5)
Middle: upper secondary	11 355 (47.2)	96 181 (40.0)	10 354 (38.9)	100 482 (37.8)
Low: elementary, ≤9 y compulsory	5032 (20.9)	31 400 (13.1)	6081 (22.9)	45 333 (17.0)
Missing data	2349 (9.8)	18 459 (7.7)	2953 (11.1)	25 806 (9.7)
Coexisting diagnoses				
*ICD-10* Chapter XVII: Chromosomal	4975 (20.7)	15 500 (6.4)	9211 (34.6)	15 867 (6.0)
Epilepsy	3809 (15.8)	2729 (1.1)	9772 (36.7)	3344 (1.3)
ASD and/or ADHD	8508 (35.4)	6744 (2.8)	7030 (26.4)	5792 (2.2)
Depression and/or anxiety	6199 (25.8)	15 302 (6.4)	3359 (12.6)	17 356 (6.5)

Abbreviations: ADHD, attention-deficit/hyperactivity disorder; ASD, autism spectrum disorder; *ICD-10 *Chapter XVII: Chromosomal*, International Statistical Classification of Diseases and Related Health Problems, Tenth Revision,* Chapter XVII: Congenital malformations, deformations, and chromosomal abnormalities.

Both the mild ID (OR, 6.21; 95% CI, 5.79-6.66) and the moderate to profound ID (OR, 13.15; 95% CI, 12.52-13.81) groups had an increased risk of premature mortality (Table 4; eFigure 2 in the Supplement), whereas the risk was expectedly higher in the moderate to profound group. In both groups, female compared with male individuals had higher relative risks (mild ID: OR, 7.06 [95% CI, 6.34-7.86] vs 5.65 [95% CI, 5.16-6.20]; moderate to profound ID: OR, 16.29 [95% CI, 15.06-17.61] vs 11.35 [95% CI, 10.66-12.09]), but the absolute risk was similar (mild ID: 7.53% vs 7.47%; moderate to profound ID: 19.34% vs 18.93%). The adjustments for the potentially confounding factors had marginal effects on the risk estimates with the exception of epilepsy (overall mortality adjusted for epilepsy, mild ID: OR, 5.43 [95% CI, 5.04-5.86]; moderate to profound ID: OR, 8.94 [95% CI, 8.42-9.48]).

**Table 4.** Risk of All-Cause, Cause-Specific, and Potentially Avoidable Mortality in Cohort 2: Individuals With Mild or Moderate to Profound Intellectual Disability (ID)^a^

Category	Cohort 2: ID severity level	Cohort 2: No./total No. (%)	Matched reference cohort, No./total No. (%)	OR (95% CI)						
				Crude	Analyses adjusted to potentially moderating factors					Adjusted for all potentially confounding factors
					Parental educational level	Congenital malformations	Epilepsy	ASD and/or ADHD	Depression and/or anxiety	
**Overall or all-cause mortality **										
All chapters	Mild ID	1803/24 059 (7.5)	4923/240 590 (2.1)	6.21 (5.79-6.66)	6.13 (5.71-6.57)	6.02 (5.61-6.46)	5.43 (5.04-5.86)	6.37 (5.93-6.85)	5.46 (5.07-5.88)	4.84 (4.46-5.24)
All chapters	Mild ID (males)	1019/13 649 (7.5)	3042/136 490 (2.2)	5.65 (5.16-6.20)	5.55 (5.05-6.09)	5.48 (4.99-6.01)	4.89 (4.42-5.40)	5.81 (5.28-6.40)	5.04 (4.58-5.55)	4.41 (3.97-4.90)
All chapters	Mild ID (females)	784/10 410 (7.5)	1881/104 100 (1.8)	7.06 (6.34-7.86)	7.01 (6.29-7.82)	6.86 (6.15-7.66)	6.29 (5.60-7.06)	7.21 (6.45-8.05)	6.11 (5.45-6.86)	5.51 (4.87-6.25)
All chapters	Moderate to profound	5081/26 602 (19.1)	8525/266 020 (3.2)	13.15 (12.52-13.81)	13.13 (12.50-13.80)	11.36 (10.79-11.97)	8.94 (8.42-9.48)^b^	14.40 (13.68-15.15)	13.03 (12.41-13.69)	8.36 (7.84-8.91)
All chapters	Moderate to profound (males)	2903/15 339 (18.9)	5584/153 390 (3.6)	11.35 (10.66-12.09)	11.31 (10.61-12.05)	9.86 (9.23-10.55)	7.93 (7.34-8.56)^b^	12.70 (11.89-13.57)	11.13 (10.45-11.86)	7.66 (7.06-8.32)
All chapters	Moderate to profound (females)	2178/11 263 (19.3)	2941/112 630 (2.6)	16.29 (15.06-17.61)	16.30 (15.07-17.63)	14.03 (12.91-15.25)	10.60 (9.65-11.64)^b^	17.20 (15.87-18.64)	16.34 (15.11-17.68)	9.44 (8.54-10.43)
**Cause-specific mortality by *ICD-10* chapter**										
Chapter I: Infections	Mild	38/24 059 (0.2)	58/240 590 (0.0)	6.55 (4.35-9.86)	6.52 (4.32-9.85)	6.59 (4.35-9.99)	6.77 (4.36-10.50)	6.40 (4.23-9.69)	6.38 (4.14-9.82)	6.57 (4.11-10.50)
	Moderate to profound	146/26 602 (0.6)	105/266 020 (0.0)	14.09 (10.95-18.14)	14.07 (10.91-18.14)	11.72 (8.94-15.38)	11.87 (8.69-16.22)	14.99 (11.58-19.40)	14.14 (10.97-18.23)	9.87 (7.10-13.72)
Chapter II: Neoplasms	Mild	293/24 059 (1.2)	1791/240 590 (0.7)	1.72 (1.51-1.96)	1.71 (1.50-1.95)	1.70 (1.49-1.94)	1.59 (1.38-1.83)	1.76 (1.54-2.00)	1.70 (1.48-1.94)	1.59 (1.38-1.84)
	Moderate to profound	561/26 602 (2.1)	3053/266 020 (1.2)	1.95 (1.77-2.14)	1.94 (1.76-2.13)	1.93 (1.75-2.14)	1.54 (1.37-1.74)	1.96 (1.78-2.16)	1.93 (1.76-2.13)	1.56 (1.39-1.76)
Chapter III: Blood, immune mechanisms	Mild	11/24 059 (0.1)	10/240 590 (0.0)	11.00 (4.67-25.90)	11.62 (4.88-27.68)	10.46 (4.37-25.01)	9.11 (3.52-23.56)	10.64 (4.41-25.63)	8.92 (3.51-22.65)	7.10 (2.63-19.17)
	Moderate to profound	25/26 602 (0.1)	25/266 020 (0.0)	10.00 (5.74-17.41)	9.89 (5.66-17.30)	8.97 (4.93-16.31)	4.09 (1.82-9.22)	11.79 (6.65-20.90)	10.73 (6.15-18.73)	3.64 (1.57-8.45)
Chapter IV: Endocrine	Mild	84/24 059 (0.4)	149/240 590 (0.1)	5.74 (4.39-7.53)	5.73 (4.36-7.53)	5.66 (4.30-7.45)	5.27 (3.91-7.10)	5.69 (4.32-7.48)	5.15 (3.86-6.88)	4.68 (3.42-6.40)
	Moderate to profound	202/26 602 (0.8)	213/266 020 (0.1)	9.72 (8.00-11.80)	9.69 (7.97-11.79)	9.28 (7.56-11.39)	8.97 (7.07-11.39)	10.60 (8.69-12.94)	9.72 (7.99-11.83)	8.96 (7.00-11.47)
Chapter V: Mental and behavioral	Mild	60/24 059 (0.3)	131/240 590 (0.1)	4.71 (3.45-6.42)	4.53 (3.32-6.19)	4.88 (3.57-6.68)	3.92 (2.77-5.57)	4.93 (3.60-6.75)	3.36 (2.40-4.72)	3.17 (2.18-4.61)
	Moderate to profound	266/26 602 (1.0)	188/266 020 (0.1)	15.13 (12.48-18.35)	15.23 (12.55-18.49)	15.36 (12.56-18.79)	10.46 (8.16-13.40)	15.04 (12.32-18.35)	14.75 (12.15-17.91)	9.64 (7.40-12.57)
Chapter VI: Nervous system	Mild	142/24 059 (0.6)	152/240 590 (0.1)	9.42 (7.49-11.85)	9.57 (7.58-12.07)	8.90 (7.04-11.26)	4.58 (3.45-6.08)^b^	9.15 (7.22-11.60)	10.29 (8.10-13.08)	4.55 (3.39-6.09)
	Moderate to profound	878/26 602 (3.3)	277/266 020 (0.1)	33.34 (29.04-38.29)	33.83 (29.44-38.88)	30.64 (26.50-35.43)	13.19 (11.02-15.80)^b^	37.16 (32.24-42.83)	34.96 (30.43-40.16)	13.70 (11.34-16.54)
Chapter IX: Circulatory system	Mild	535/24 059 (2.2)	1287/240 590 (0.5)	5.25 (4.69-5.88)	5.24 (4.67-5.87)	5.17 (4.60-5.80)	5.00 (4.42-5.66)	5.24 (4.67-5.88)	4.74 (4.20-5.35)	4.44 (3.91-5.06)
	Moderate to profound	1020/26 602 (3.8)	2199/266 020 (0.8)	5.80 (5.33-6.30)	5.73 (5.28-6.23)	5.66 (5.19-6.17)	5.47 (4.95-6.05)	5.79 (5.32-6.31)	5.64 (5.19-6.14)	5.08 (4.58-5.63)
Chapter X: Respiratory	Mild	158/24 059 (0.7)	216/240 590 (0.1)	7.94 (6.41-9.82)	7.73 (6.24-9.58)	8.07 (6.51-10.02)	6.87 (5.44-8.69)	7.98 (6.44-9.89)	6.87 (5.47-8.63)	6.06 (4.76-7.70)
	Moderate to profound	547/26 602 (2.1)	375/266 020 (0.1)	15.87 (13.84-18.18)	15.65 (13.64-17.95)	13.86 (11.99-16.01)	13.41 (11.37-15.82)	16.36 (14.24-18.81)	15.85 (13.82-18.19)	11.60 (9.76-13.80)
Chapter XI: Digestive	Mild	68/24 059 (0.3)	188/240 590 (0.1)	3.71 (2.80-4.92)	3.57 (2.69-4.74)	3.61 (2.72-4.80)	3.34 (2.45-4.54)	3.74 (2.80-4.98)	3.46 (2.57-4.67)	3.06 (2.22-4.22)
	Moderate to profound	263/26 602 (1.0)	324/266 020 (0.1)	8.42 (7.13-9.93)	8.45 (7.16-9.99)	7.34 (6.15-8.75)	6.61 (5.36-8.16)	8.63 (7.29-10.22)	8.46 (7.16-10.00)	6.10 (4.89-7.60)
Chapter XII: Skin	Moderate to profound	7/26 602 (0.0)	5/266 020 (0.0)	14.00 (4.44-44.11)	16.57 (5.21-52.70)	12.30 (3.69-40.94)	9.32 (2.26-38.49)	15.32 (4.80-48.89)	12.22 (3.81-39.21)	9.54 (1.98-45.93)
Chapter XIII: Musculoskeletal	Mild	7/24 059 (0.0)	33/240 590 (0.0)	2.13 (0.94-4.84)	2.13 (0.93-4.85)	2.10 (0.92-4.82)	1.94 (0.77-4.85)	2.14 (0.93-4.95)	2.22 (0.93-5.25)	2.21 (0.83-5.87)
	Moderate to profound	51/26 602 (0.2)	40/266 020 (0.0)	12.75 (8.43-19.29)	12.56 (8.28-19.05)	10.52 (6.76-16.37)	10.04 (6.13-16.46)	12.95 (8.49-19.75)	12.92 (8.52-19.60)	7.74 (4.55-13.17)
Chapter XIV: Genitourinary	Mild	31/24 059 (0.1)	29/240 590 (0.0)	11.19 (6.67-18.77)	11.61 (6.87-19.62)	10.77 (6.34-18.28)	9.47 (5.42-16.54)	11.19 (6.67-18.77)	12.04 (7.02-20.66)	9.12 (5.04-16.52)
	Moderate to profound	93/26 602 (0.4)	51/266 020 (0.0)	18.80 (13.30-26.58)	19.21 (13.57-27.21)	18.86 (13.15-27.05)	18.79 (12.52-28.20)	18.14 (12.78-25.77)	18.70 (13.19-26.51)	16.59 (10.85-25.34)
Chapter XVII: Malformations, chromosomal	Mild	38/24 059 (0.2)	20/240 590 (0.0)	19.00 (11.06-32.65)	19.69 (11.30-34.30)	7.62 (4.34-13.36)	18.15 (10.11-32.58)	19.49 (11.13-34.13)	21.48 (12.29-37.53)	7.97 (4.16-15.27)
	Moderate to profound	548/26 602 (2.1)	46/266 020 (0.0)	121.66 (89.77-164.87)	125.38 (92.44-170.05)	39.45 (28.64-54.35)^b^	81.81 (58.71-114.00)	152.68 (112.12-207.92)	128.98 (95.15-174.83)	34.06 (23.72-48.90)
Chapter XVIII: Symptoms, signs	Mild	36/24 059 (0.2)	84/240 590 (0.0)	4.34 (2.93-6.44)	4.16 (2.80-6.18)	4.49 (3.01-6.68)	4.01 (2.60-6.20)	4.55 (3.04-6.80)	4.20 (2.77-6.36)	4.23 (2.68-6.68)
	Moderate to profound	75/26 602 (0.3)	156/266 020 (0.1)	4.85 (3.68-6.39)	4.83 (3.66-6.38)	4.61 (3.43-6.18)	3.83 (2.67-5.51)	4.76 (3.57-6.34)	4.55 (3.44-6.01)	3.69 (2.53-5.39)
Chapter XIX-XX: External causes	Mild	293/24 059 (1.2)	768/240 590 (0.3)	3.93 (3.43-4.51)	3.78 (3.29-4.35)	3.89 (3.39-4.47)	4.09 (3.54-4.74)	3.82 (3.30-4.42)	2.75 (2.37-3.19)	2.97 (2.53-3.49)
	Moderate to profound	301/26 602 (1.1)	1445/266 020 (0.5)	2.11 (1.86-2.39)	2.05 (1.80-2.32)	2.28 (2.00-2.60)	2.17 (1.87-2.53)	2.09 (1.83-2.38)	1.86 (1.64-2.11)	2.14 (1.83-2.52)
**Potentially avoidable mortality**^c^										
Potentially avoidable, both amenable and preventable	Mild	940/24 059 (3.9)	2788/240 590 (1.2)	4.20 (3.86-4.57)	4.12 (3.78-4.48)	4.11 (3.78-4.48)	3.69 (3.37-4.04)	4.15 (3.80-4.53)	3.46 (3.17-3.78)	3.09 (2.81-3.41)
	Moderate to profound	1747/26 602 (6.6)	4719/266 020 (1.8)	4.64 (4.36-4.93)	4.58 (4.30-4.88)	4.43 (4.15-4.73)	3.53 (3.27-3.81)	4.60 (4.31-4.90)	4.38 (4.11-4.66)	3.27 (3.01-3.55)
Amenable to health care intervention	Mild	663/24 059 (2.8)	1601/240 590 (0.7)	5.20 (4.70-5.76)	5.15 (4.65-5.71)	5.12 (4.62-5.68)	4.29 (3.83-4.80)	5.18 (4.67-5.75)	4.65 (4.17-5.18)	3.85 (3.43-4.34)
	Moderate to profound	1426/26 602 (5.4)	2760/266 020 (1.0)	6.65 (6.19-7.15)	6.61 (6.15-7.11)	6.13 (5.68-6.62)	4.91 (4.49-5.38)	6.57 (6.10-7.08)	6.50 (6.04-6.99)	4.46 (4.06-4.90)
Preventable	Mild	640/24 059 (2.7)	2318/240 590 (1.0)	3.14 (2.85-3.45)	3.07 (2.79-3.38)	3.09 (2.81-3.40)	3.04 (2.75-3.37)	3.07 (2.78-3.39)	2.49 (2.25-2.75)	2.48 (2.22-2.76)
	Moderate to profound	897/26 602 (3.4)	3895/266 020 (1.5)	2.51 (2.32-2.71)	2.47 (2.29-2.67)	2.56 (2.36-2.78)	2.29 (2.09-2.52)	2.48 (2.29-2.68)	2.32 (2.15-2.51)	2.20 (1.99-2.43)

Abbreviations: ADHD, attention-deficit/hyperactivity disorder; ASD, autism spectrum disorder; *ICD-10, International Statistical Classification of Diseases and Related Health Problems, Tenth Revision*; OR, odds ratio.

^a^ Categories with fewer than 5 individuals in any of the cells are not shown. The detailed description of the confounders, including diagnostic codes, is provided in eTable 1 in the Supplement.

^b^ Categories show significant attenuation of risk after adjustment for epilepsy and stratified for epilepsy diagnostic status in eTable 6 in the Supplement.

^c^ Definition is from the Office for National Statistics.^29^

In cohort 2, individuals with mild ID or moderate to profound ID had significantly increased risk in all categories that could be analyzed, and the overall pattern of results showed generally higher risks in the moderate to profound ID group (Table 4). Thus, among the 15 analyzed cause-of-death categories, those with moderate to profound ID had higher risk in 9 categories and similar risk in 5 categories compared with those with mild ID. External causes of death was the only category in which individuals with mild ID (OR, 3.93; 95% CI, 3.43-4.51) had a higher risk than those with moderate to profound ID (OR, 2.11; 95% CI, 1.86-2.39). The 3 most common causes of death within each category are depicted in eTable 4 in the Supplement, and stratified analyses with or without epilepsy are shown in eTable 7 in the Supplement. As such, ID was identified as a primary cause of death for 13 of 1803 individuals (0.7%) with mild ID and among 130 of 5081 individuals (2.6%) with moderate to profound ID (eTable 5 in the Supplement).

The risk of possibly avoidable deaths with causes that were amenable to health care interventions was highest among individuals with mild ID (OR, 5.20; 95% CI, 4.70-5.76) and those with moderate to profound ID (OR, 6.65; 95% CI, 6.19-7.15). The risk of preventable deaths was also significantly higher for those with mild ID (OR, 3.14; 95% CI, 2.85-3.45) and those with moderate to profound ID (OR, 2.51; 95% CI, 2.32-2.71) (Table 4).

## Discussion

This nationwide population-based cohort study suggests that health challenges remain for people with ID, including mild ID, in a contemporary Swedish welfare society. A large proportion of the excess risk for premature mortality in those with mild ID was classified as potentially treatable. Expectedly, increased severity of ID was associated with considerably higher mortality risk. Common causes of death (neoplasms and diseases of the circulatory system) were more frequent among individuals with ID. Moreover, diseases of the nervous system, especially epilepsy, were frequently observed both as a cause of death and as a confounding factor in several cause-of-death categories.

### All-Cause Mortality

We found an increased mortality risk in the 2 cohorts, including 2.86-fold risk in young adults with mild ID. These results are in line with those in other contemporary studies, although previous studies were mostly not stratified for ID severity level or only included small samples of mild ID.^5,9,10,11,13,30,31^ Nevertheless, these results suggest that, even in a modern egalitarian welfare society, health challenges remain for young adults with ID who took part in adjusted education.

Both syndromal ID and differences in life experiences (eg, living arrangements, community participation, and access to support) might play a role in the large differences in premature mortality among those with different ID severity levels (cohort 2). Thus, in accordance to a previous smaller study,^5^ we observed a 15-year gap (almost 1 SD) in longevity in individuals with moderate to profound ID vs their matched reference cohort and a 5-year gap (one-third of an SD) between those with mild ID vs their matched reference cohort.

The large sample size and access to high-quality registries in this study allowed us to analyze several potential confounders. Similar to the findings in other studies,^5,6,9,11,14^ female sex was associated with higher relative risk for overall mortality in cohorts 1 and 2 (vs same-sex reference individuals in the matched reference cohorts), whereas the observation of a similar proportion of deaths in both sexes in cohorts 1 and 2 was in line with the results of a recent study from Australia.^32^ Moreover, confounders had only a limited association with all-cause mortality. Thus, we could not confirm in this study the recently reported higher mortality risk in socioeconomically disadvantaged people with ID^32^ by using parental educational level as a proxy for socioeconomic status.

### Cause-Specific Mortality

The risks of premature mortality in circulatory diseases and neoplasms were higher among people with ID compared with the matched reference cohorts. The current study does not explain the reasons for the increased risks; however, previous studies suggest a higher prevalence of risk factors, such as obesity, hypertension, and diabetes, among individuals with ID.^33^ Despite the benefits of deinstitutionalization, challenges may exist pertaining to a lower restriction in living arrangement and an associated increase in behavioral factors, such as smoking, sedentary lifestyle, and poor diet.^8^

Deaths attributed to nervous system diseases (eg, epilepsy) have consistently been found to be more prevalent among individuals with ID,^6,9,10,12,15^ and the results were similar in this study. Generally, epilepsy was indicated both as a major confounder in several categories for cohort 2 and as a common primary cause of death. National clinical guidelines for different ID severity levels that address multimorbidity and possibly avoidable deaths^6^ are still lacking in Sweden. Similarly, improved primary care for people with ID that includes scheduled health checks^34^ and health action plans remains to be implemented on a national level. To promote preventive health interventions and to reduce diagnostic overshadowing, which has been associated with late identification and treatment of ill health, educating the housing support staff may be needed.^35^

We observed an increased risk of death from respiratory diseases (eg, pneumonitis from foods or fluids and pneumonia) in cohort 2, and this risk was higher among individuals with moderate to profound ID (ie, almost as common a cause of death as neoplasms), a finding that has been found in some^9,10,12,15^ but not all^6^ studies. Dysphagia (difficulties with eating, drinking, or swallowing) is common, especially in individuals with more severe levels of ID with coexisting motor impairment.^36^ Dysphagia is associated with poor nutritional status, asphyxiation, respiratory infections, and premature mortality.^37^ The identified knowledge gaps pertain to clinical management, including assessment, mealtime support, positioning, dietary modification, and well-being outcome.^36,37^ In addition to respiratory diseases, we identified several cases that may be related to dysphagia among external causes of death (inhalation and ingestion of food that cause obstruction in the respiratory tract).

The moderate to profound group had a higher risk in several cause-of-death categories compared with the mild ID group in cohort 2. However, those with mild ID had a higher risk in the external cause-of-death category, an important topic for further research to better understand and prevent premature mortality attributed to external causes.

### Potentially Avoidable Mortality

Among the young adults with mild ID (cohort 1), most deaths were attributed to potentially avoidable mortality,^29^ with an especially high risk of death attributable to causes that were amenable to health care intervention, as has been previously suggested.^6,10^ This obvious health challenge, which is indicative of persistent inequality in health care encounters for people with ID, is shown in the Figure. A high proportion (55%) of the amenable mortality among young adults with mild ID was associated with epilepsy.

**Figure.**
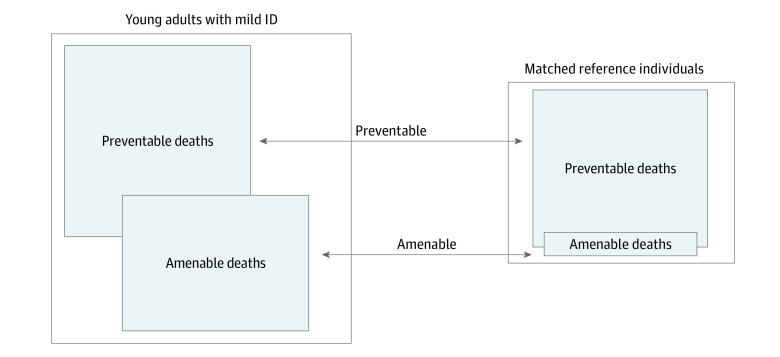
Overall Mortality and Potentially Avoidable Mortality in Young Adults With Mild Intellectual Disability (ID) Proportional area chart shows higher overall mortality rate among young adults (born 1980-1991) with mild ID (88.6 per 10 000) compared with the matched reference cohort (31.3 per 10 000) as well as between-group comparisons and within-group proportions of the deaths categorized as possibly preventable (36.7% vs 78.5%; odds ratio [OR], 1.32; 95% CI, 0.97-1.81) or amenable to health care interventions (25.8% vs 9.9%; OR, 7.75; 95% CI, 4.85-12.39).

The high risk of amenable mortality may be associated with both environmental factors (eg, accessibility of high-quality health care and reliance on social networks) and ID-related difficulties during health encounters. Health advocacy programs for individuals with ID may support their comprehension and communication, improve documentation of health encounters and use of preventive health screening programs^35,38^ and primary care,^39,40^ and promote active participation in health encounters. Heavy reliance on family members for both advocacy and care is common,^35,41^ but health care encounters must address family needs in addition to assessing and making reasonable adjustments to the needs of the individual with ID.

We believe that, ultimately, this study informs the persistent health challenges and the high burden of disease in ID.^42^ Clear identification of people with ID and well-defined causes of death in national health registers are fundamental to creating optimal conditions for research and subsequent services. National clinical guidelines may improve the health system for this patient population. Such guidelines may cover increasing awareness among health care professionals and implementation of scheduled health checks, thus helping to reduce health inequality and excess mortality in people with ID.

### Limitations

This study has several limitations. First, despite being widely included in available national registers, ID may remain unrecognized in some individuals. Moreover, given that the assignment to a specific USSID program is based on the individual functional profile, which is created from assessments by multiprofessional teams rather than specific *ICD* codes, some misclassification occurs, especially regarding individuals with moderate ID. Second, Sweden has a tax-funded health care system with universal access, and the generalizability of these results to other countries may be limited by societal differences in welfare policies, practices, and resources.^7,43^ Third, we lacked information on the social network, an especially important health factor for individuals with ID. Family members and social networks may be advocates for pursuing their rights, supporting independent living,^44^ and reducing loneliness, which may have an association with overall mortality.^45^

## Conclusions

This cohort study found excess premature mortality and high risk of deaths with causes that were amenable to health care intervention among individuals with ID, suggesting the persistent health challenges and inequality in health care encounters that this patient population grapples with in a contemporary welfare society. Establishing national clinical guidelines may improve the health system for people with ID, including mild ID.

## Notes

Supplement. **eTable 1.** ICD-Codes Used to Define Intellectual Disability With Different Severity Levels, and Comorbid Conditions in the Adjusted Analyses (Potentially Confounding Factors), As Well As Other Than ICD Code Based Categorization, Such As Parental Education Level **eTable 2.** Descriptive Statistics Stratified According to the USSID Programs (from the HURPID Register), As Well As the Correspondence of the USSID Program Allocation to the NPR Diagnoses **eTable 3.** Definitions of Primary (All-Cause Mortality) and Secondary (Cause-Specific Mortality and Potentially Avoidable Mortality) Outcomes **eTable 4.** The Three Most Common Diagnoses (More Than Three Given if the Number of Individuals Was Equal Across Several Diagnoses) in the Specific Causes of Death Categories in which ID Cohort 1 (Young Adults With Mild ID Born 1980-1990) Had Higher Risk Than Matched Reference Individuals from the General Population **eTable 5.** The Three Most Common Diagnoses in the Specific Causes of Death Categories in which ID Cohort 2 (Identified from the National Patient Register (NPR) Born 1932-2013, All Ages, All ID Severity Levels) Had Higher Mortality Compared than the Matched Reference Individuals from the Total Population Register **eTable 6.** The Most Common Diagnoses Defined as Potentially Avoidable Deaths (Both Preventable and Amenable), Potentially Preventable Deaths and Deaths Potentially Amenable to Health Care (Definitions Shown in eTable 3), in ID Cohort 1 Young Adults With Mild ID and the Matched Reference Individuals, as well as ID Cohort 2 All Cases with ID from the National Patient Register and Their Reference Group **eTable 7.** Post Hoc Analyses of ID cohort 2, Stratified According to Diagnostic Status of Co-morbid Epilepsy (Yes/No) for Overall and Cause-Specific Mortality, Respectively **eFigure 1.** Flowchart of the Two Included Cohorts **eFigure 2.** Kaplan-Meier Survival Estimators
