# Supplementary material for: Association of Intellectual Disability With All-Cause and Cause-Specific Mortality in Sweden
Source: JAMA Netw Open. 2021 Jun 22;4(6):e2113014. doi: 10.1001/jamanetworkopen.2021.13014 (PMC8220491; doi:10.1001/jamanetworkopen.2021.13014)
Supplement: Supplement. — eTable 1. ICD-Codes Used to Define Intellectual Disability With Different Severity Levels, and Comorbid Conditions in the Adjusted Analyses (Potentially Confounding Factors), As Well As Other Than ICD Code Based Categorization, Such As Parental Education Level eTable 2. Descriptive Statistics Stratified According to the USSID Programs (from the HURPID Register), As Well As the Correspondence of the USSID Program Allocation to the NPR Diagnoses eTable 3. Definitions of Primary (All-Cause Mortality) and Secondary (Cause-Specific Mortality and Potentially Avoidable Mortality) Outcomes eTable 4. The Three Most Common Diagnoses (More Than Three Given if the Number of Individuals Was Equal Across Several Diagnoses) in the Specific Causes of Death Categories in which ID Cohort 1 (Young Adults With Mild ID Born 1980-1990) Had Higher Risk Than Matched Reference Individuals from the General Population eTable 5. The Three Most Common Diagnoses in the Specific Causes of Death Categories in which ID Cohort 2 (Identified from the National Patient Register (NPR) Born 1932-2013, All Ages, All ID Severity Levels) Had Higher Mortality Compared than the Matched Reference Individuals from the Total Population Register eTable 6. The Most Common Diagnoses Defined as Potentially Avoidable Deaths (Both Preventable and Amenable), Potentially Preventable Deaths and Deaths Potentially Amenable to Health Care (Definitions Shown in eTable 3), in ID Cohort 1 Young Adults With Mild ID and the Matched Reference Individuals, as well as ID Cohort 2 All Cases with ID from the National Patient Register and Their Reference Group eTable 7. Post Hoc Analyses of ID cohort 2, Stratified According to Diagnostic Status of Co-morbid Epilepsy (Yes/No) for Overall and Cause-Specific Mortality, Respectively eFigure 1. Flowchart of the Two Included Cohorts eFigure 2. Kaplan-Meier Survival Estimators [file jamanetwopen-e2113014-s001.pdf]

Supplementary Online Content

Hirvikoski T, Boman M, Tideman M, Lichtenstein P, Butwicka A. Association of intellectual disability with all-cause and cause-specific mortality in Sweden. *JAMA Netw Open*. 2021;4(6):e2113014. doi:10.1001/jamanetworkopen.2021.13014

eTable 1. ICD-Codes Used to Define Intellectual Disability With Different Severity Levels, and Comorbid Conditions in the Adjusted Analyses (Potentially Confounding Factors), As Well As Other Than ICD Code Based Categorization, Such As Parental Education Level

eTable 2. Descriptive Statistics Stratified According to the USSID Programs (from the HURPID Register), As Well As the Correspondence of the USSID Program Allocation to the NPR Diagnoses

eTable 3. Definitions of Primary (All-Cause Mortality) and Secondary (Cause-Specific Mortality and Potentially Avoidable Mortality) Outcomes

eTable 4. The Three Most Common Diagnoses (More Than Three Given if the Number of Individuals Was Equal Across Several Diagnoses) in the Specific Causes of Death Categories in which ID Cohort 1 (Young Adults With Mild ID Born 1980-1990) Had Higher Risk Than Matched Reference Individuals from the General Population

eTable 5. The Three Most Common Diagnoses in the Specific Causes of Death Categories in which ID Cohort 2 (Identified from the National Patient Register (NPR) Born 1932-2013, All Ages, All ID Severity Levels) Had Higher Mortality Compared than the Matched Reference Individuals from the Total Population Register

eTable 6. The Most Common Diagnoses Defined as Potentially Avoidable Deaths (Both Preventable and Amenable), Potentially Preventable Deaths and Deaths Potentially Amenable to Health Care (Definitions Shown in eTable 3), in ID Cohort 1 Young Adults With Mild ID and the Matched Reference Individuals, as well as ID Cohort 2 All Cases with ID from the National Patient Register and Their Reference Group

© 2021 Hirvikoski T et al. *JAMA Network Open.*

eTable 7. Post Hoc Analyses of ID cohort 2, Stratified According to Diagnostic Status of Co-morbid Epilepsy (Yes/No) for Overall and Cause-Specific Mortality, Respectively

eFigure 1. Flowchart of the Two Included Cohorts

eFigure 2. Kaplan-Meier Survival Estimators

This supplementary material has been provided by the authors to give readers additional information about their work.

© 2021 Hirvikoski T et al. *JAMA Network Open.*

eTable 1. ICD-Codes Used to Define Intellectual Disability With Different Severity Levels, and Comorbid Conditions in the Adjusted Analyses (Potentially Confounding Factors), As Well As Other Than ICD Code Based Categorization, Such As Parental Education Level

**Variable**	**Categorization based on the International Classification of Diseases (ICD), ascertained in the National Patient Register (NPR)**			**Other than ICD code based categorization**
	**ICD-8**	**ICD-9**	**ICD-10**	
	**(1969-1986)**	**(1987-1996)**	**(1997-)**	
Intellectual disability, all	311-315	317-319	F70-F79	
**Categorization of to mild and more severe intellectual disability (ID)**				
Mild ID	311	317	F70	In ID cohort 1 mild ID, attendance to the following USSID programs, identified from the HURPID register: National (including Special programs); Individual /Vocational program; incomplete grades.
More severe ID				
Moderate	312	318A	F71	
Severe	313	318B	F72	
Profound	314	318C	F73	
Unspecified	315	319	F78; F79	

© 2021 Hirvikoski T et al. *JAMA Network Open.*

Variable	Categorization based on the International Classification of Diseases (ICD), ascertained in the National Patient Register (NPR)			Other than ICD code based categorization
**Chormosomal abnormalities and epilepsy**				
ICD-10 chapter XVII Congenital malformation and chromosomal abnormalities	740-759	740-759	Q00-Q99	
Epilepsy	345	345	G40.00 – G41.9.	
**Other neurodevelopmental disorders**				
ADHD		314J; 314W; 314X	F90	
Autism spectrum disorders	-	299A; 299B; 299W; 299X	F84.0; F84.1; F84.3; F84.5; F84.8; F84.9	
**Psychiatric comorbidity**				
Anxiety or depression	296.2; 298.0; 300.0; 300.2; 300.4	296B; 298A; 300A; 300C; 300E, 311	F32; F33; F40 – F41	
**Family/parent education level**				
Education level				The highest education level [elementary, secondary, higher] held by either of the biological parents during life-time. The data was extracted from the population and housing census (for years 1970-1984); RAMS (for years 1985-1989) and LISA (for years 1990-2013).

© 2021 Hirvikoski T et al. *JAMA Network Open.*

Note: HURPID: The Halmstad University Register on Pupils with Intellectual Disability; RAMS: Register based labour market statistics (RAMS by Swedish acronym); LISA a longitudinal integration database for health insurance and labour market studies (LISA by Swedish acronym).

© 2021 Hirvikoski T et al. *JAMA Network Open.*

eTable 2. Descriptive Statistics Stratified According to the USSID Programs (from the HURPID Register), As Well As the Correspondence of the USSID Program Allocation to the NPR Diagnoses

**Characteristics**	**USSID - Program**<br><br>**Number (%) or mean years (SD) (min-max)**			
	**Individual program/activity training**		**National programs including Special programs, Individual program/Vocational training, Incomplete**	
	**Cases from the HURPID**	**Controls**	**Cases from the HURPID**	**Controls**
Total n, born 1980-1991	1,193	11,930	11,320	113,200
Males	691 (58.0)	6,910 (58.0)	6,642 (58.7)	66,420 (58.7)
Females	502 (42.0)	5,020 (42.0)	4,678 (41.3)	46,780 (41.3)
Age at finishing the USSID	20.9 (0.6) (18.3-23.7)	N/A	20.6 (0.7) (18.0-24.0)	N/A
Identified in the NPR with moderate, severe or profound ID	582 (48.8)	N/A	830 (7.3)	N/A
Identified in the NPR with mild ID	59 (4.9)	N/A	2,789 (24.7)	N/A
Identified in the NPR with unspecified ID	186 (15.6)	N/A	627 (5.5)	N/A
Not identified in the NPR (missing)	366 (30.7)	N/A	7,074 (62.5)	N/A
Deaths	52 (4.4)	38 (0.3)	83 (0.7)	364 (0.3)

© 2021 Hirvikoski T et al. *JAMA Network Open.*

Note: Cases: Included in HURPID, Controls: Not included in HURPID and not affected by Intellectual Disability in NPR, HURPID: Halmstad University Register on Pupils with Intellectual Disability, NPR: National Patient Register, %: Percent, SD: Standard Deviation, min: Minimum, max: Maximum, Activity: Activity training, National: National Programmes, Special: Specially designed Programmes, Vocational: Vocational training, Incomplete: Incomplete certificate, N/A: Not applicable.

© 2021 Hirvikoski T et al. *JAMA Network Open.*

eTable 3. Definitions of Primary (All-Cause Mortality) and Secondary (Cause-Specific Mortality and Potentially Avoidable Mortality) Outcomes

**Primary outcome: overall / all-cause mortality**		
All ICD-10 chapters		
**Secondary outcome 1. Cause specific mortality categorized according to the ICD-10 chapters**		
Chapter I	***Infections (ICD codes A00-B99)***	Certain infectious and parasitic diseases that are generally recognized as communicable or transmissible, such as tuberculosis and viral infections of the central nervous system.
Chapter II	***Neoplasms (C00-D48)***	Both malignant and benign neoplasms, as well as cancer diseases of uncertain or unknown behavior.
Chapter III	***Diseases of the blood and blood-forming organs (D50-D89)***	Diseases of the blood and blood-forming organs and certain disorders involving the immune mechanism.
Chapter IV	***Endocrine, nutritional and metabolic diseases (E00-E99)***	E.g. diabetes mellitus, disorders of thyroid gland, malnutrition, and obesity.
Chapter V	***Mental and behavioral disorders (F00-F99)***	Psychiatric disorders, organic mental disorders (e.g. vascular dementia), and drug- and alcohol intoxication.
Chapter VI	***Diseases of nervous system (G00-G99)***	Systemic atrophies of CNS, degenerative diseases of the nervous system (for example Alzheimer's disease) and epilepsy.
Chapter IX	***Diseases of the circulatory system (I00-I99)***	Ischaemic, pulmonary, or hypertensive heart diseases, as well as cerebrovascular diseases and other disorders of the circulatory system.
Chapter X	***Diseases of the respiratory system (J00-J99)***	E.g. influenza and pneumonia, as well as lung diseases due to external agents.
Chapter XI	***Diseases of the digestive system (K00-K99)***	Diseases of stomach, intestines, liver, gallbladder, and pancreas, as well as other diseases of the digestive system.
Chapter XII	***Diseases of the skin and subcutaneous tissue (L00-L99)***	E.g. Infections of the skin and subcutaneous tissue.
**Secondary outcome 1. Cause specific mortality categorized according to the ICD-10 chapters**		
Chapter XIII	***Diseases of the musculoskeletal system and connective tissue (M00-M99)***	

© 2021 Hirvikoski T et al. *JAMA Network Open.*

Chapter XIV	***Diseases of the genitourinary system (N00-N99)***	E.g. renal failure, other disorders of kidney and ureter as well as diseases of urinary system.
Chapter XVII	***Congenital malformations (Q00-Q99)***	Congenital malformations and deformations (of for example nervous, respiratory or circulatory system), as well as chromosomal abnormalities, not elsewhere classified.
Chapter XVIII	***Symptoms, signs and abnormal findings not elsewhere classified (R00-R99)***	Diagnoses based on symptoms, signs and abnormal clinical and laboratory findings, not elsewhere classified. In general, categories in this chapter include the less well-defined conditions, such as "senility" or "unspecified symptoms and signs involving the circulatory and respiratory systems"
Chapter XIX	***Injury, poisoning and certain other consequences of external causes***	Injuries related to single body regions, injuries to multiple or unspecified body regions as well as poisoning and certain other consequences of external causes.
Chapter XX	***External causes of morbidity and mortality (V01-Y98)***	Intentional self-harm/suicide, other X diagnoses than intentional self-harm (assault; accidents), Y diagnoses (event of undetermined intent; legal intervention and operations of war; complications of medical and surgical care; sequelae of external causes of morbidity and mortality; supplementary factors related to causes of morbidity and mortality classified elsewhere), V diagnoses (transport accidents), as well as W diagnoses (other external causes of accidental injury).
**Secondary outcome 2. Potentially avoidable mortality**		
Potentially avoidable, all		According to the definition of the Office for National Statistics, UK [48]. Avoidable deaths are all those defined as amenable or preventable.
- Amenable to health care		Causes of death (listed according to ICD) that are treatable, i.e. timely and effective healthcare interventions after the onset of diseases are often associated with reduced case fatality.
- Preventable		Causes of death (listed according to ICD), in which incidence can be reduces through effective public health and primary prevention interventions (i.e., before the onset of diseases or injuries).

© 2021 Hirvikoski T et al. *JAMA Network Open.*

eTable 4. The Three Most Common Diagnoses (More Than Three Given if the Number of Individuals Was Equal Across Several Diagnoses) in the Specific Causes of Death Categories in which ID Cohort 1 (Young Adults With Mild ID Born 1980-1990) Had Higher Risk Than Matched Reference Individuals from the General Population

Cause of death category (ICD-10 chapter)	The three most common causes of death in mild ID	The three most common causes of death in reference individuals
**Neoplasms**	1. Malignant neoplasm of brain<br>2-3. Malignant melanoma of skin; Malignant neoplasm of peripheral nerves and autonomic nervous system	1. Lymphoid leukemia<br>2. Myeloid leukemia<br>3-6. Malignant neoplasm of bone and articular cartilage; Malignant neoplasm of breast; Malignant neoplasm of testis; Malignant neoplasm of brain
**Diseases of nervous system**	1. Epilepsy<br>2. Primary disorders of muscles<br>3. Encephalitis, myelitis and encephalomyelitis; Other degenerative diseases of nervous system, not elsewhere classified	1. Epilepsy<br>2. Primary disorders of muscles<br>3. Other disorders of brain
**Diseases of circulatory system**	1-2. Acute myocarditis; Cardiomyopathy<br>3. Cardiac arrest; Complications and ill-defined descriptions of heart disease; Intracerebral haemorrhage.	1. Nontraumatic intracerebral haemorrhage<br>2. Cardiomyopathy<br>3-6. Pulmonary embolism; Acute myocarditis; Other cardiac arrhythmias; Nontraumatic subarachnoid haemorrhage.

© 2021 Hirvikoski T et al. *JAMA Network Open.*

eTable 5. The Three Most Common Diagnoses in the Specific Causes of Death Categories in which ID Cohort 2 (Identified from the National Patient Register (NPR) Born 1932-2013, All Ages, All ID Severity Levels) Had Higher Mortality Compared than the Matched Reference Individuals from the Total Population Register

**Cause of death category (ICD-10 chapter)**	**ID group (mild ID versus moderate to profound)**	**The three most common causes of death in mild ID**	**The three most common causes of death in reference individuals**
Certain infectious and parasitic diseases	Mild ID	1. Other sepsis<br>2. Other and unspecified infectious diseases<br>3-5. Infectious gastroenteritis and colitis, unspecified; Chronic viral hepatitis; Sequelae of tuberculosis	1. Other sepsis<br>2. Chronic viral hepatitis<br>3. Atypical virus infections of central nervous system
	Moderate to profound	1. Other sepsis<br>2. Other and unspecified infectious diseases<br>3. Infectious gastroenteritis and colitis, unspecified	1. Other sepsis<br>2. Other and unspecified infectious diseases<br>3. Chronic viral hepatitis
Neoplasms	Mild ID	1. Malignant neoplasm of bronchus and lung<br>2. Malignant neoplasm of breast<br>3. Malignant neoplasm of colon	1. Malignant neoplasm of bronchus and lung<br>2. Malignant neoplasm of breast<br>3. Malignant neoplasm of pancreas
	Moderate to profound	1. Malignant neoplasm of colon<br>2. Malignant neoplasm of breast<br>3. Malignant neoplasm of bronchus and lung	1. Malignant neoplasm of bronchus and lung<br>2. Malignant neoplasm of colon<br>3. Malignant neoplasm of pancreas
Diseases of the blood and blood-forming organs and certain disorders involving the immune mechanism	Mild ID	1 -2. Immunodeficiency with predominantly antibody defects; Sarcoidosis<br>3. Other anemias; Neutropenia; Other and unspecified diseases of blood and blood-forming organs; Immunodeficiency associated with other major defects; Other disorders involving the immune mechanism, not elsewhere classified	1-4. Other aplastic anemias; Other anemias; Neutropenia; Diseases of spleen
	Moderate to profound	1. Immunodeficiency with predominantly antibody defects<br>2. Other immunodeficiencies<br>3-4. Neutropenia; Immunodeficiency associated with other major defects	1. Purpura and other hemorrhagic conditions<br>2. Other specified diseases with participation of lymphoreticular and reticulohistiocytic tissue<br>3-5. Thalassemia; Other aplastic anemias and other bone marrow failure syndromes; Immunodeficiency with predominantly antibody defects

© 2021 Hirvikoski T et al. *JAMA Network Open.*

Cause of death category (ICD-10 chapter)	ID group (mild ID versus moderate to profound)	The three most common causes of death in mild ID	The three most common causes of death in reference individuals
Endocrine, nutritional and metabolic diseases	Mild ID	1. Unspecified diabetes mellitus<br>2. Obesity<br>3. Type 2 diabetes mellitus	1. Unspecified diabetes mellitus<br>2. Type 2 diabetes mellitus<br>3. Type 1 diabetes mellitus
	Moderate to profound	1. Unspecified diabetes mellitus<br>2. Disorders of sphingolipid metabolism and other lipid storage disorders<br>3. Other and unspecified metabolic disorders	1. Unspecified diabetes mellitus<br>2. Type 2 diabetes mellitus<br>3. Type 1 diabetes mellitus
Mental and behavioral disorders	Mild ID	1. Unspecified dementia; Unspecified intellectual disabilities*<br>2. Alcohol related disorders<br>3. Schizophrenia	1. Alcohol related disorders<br>2. Unspecified dementia<br>3. Vascular dementia
	Moderate to profound	1. Unspecified intellectual disabilities*<br>2. Profound intellectual disabilities*<br>3. Unspecified dementia	1. Alcohol related disorders<br>2. Unspecified dementia<br>3. Vascular dementia
Diseases of the nervous system	Mild ID	1. Epilepsy<br>2. Primary disorders of muscles<br>3. Cerebral palsy	1. Spinal muscular atrophy and related syndromes<br>2. Alzheimer disease<br>3. Multiple sclerosis
	Moderate to profound	1. Cerebral palsy<br>2. Epilepsy<br>3. Other disorders of brain	1. Spinal muscular atrophy and related syndromes<br>2. Alzheimer disease<br>3. Parkinson disease
Diseases of the circulatory system	Mild ID	1. Acute myocardial infarction<br>2. Chronic ischaemic heart disease<br>3. Cerebral infarction	1. Acute myocardial infarction<br>2. Chronic ischaemic heart disease<br>3. Functional disorders of polymorphonuclear neutrophils
	Moderate to profound	1. Acute myocardial infarction<br>2. Chronic ischaemic heart disease<br>3. Heart failure	1. Acute myocardial infarction<br>2. Chronic ischaemic heart disease<br>3. Functional disorders of polymorphonuclear neutrophils

© 2021 Hirvikoski T et al. *JAMA Network Open.*

Cause of death category (ICD-10 chapter)	ID group (mild ID versus moderate to profound)	The three most common causes of death in mild ID	The three most common causes of death in reference individuals
Diseases of the respiratory system	Mild ID	1. Other chronic obstructive pulmonary disease<br>2. Pneumonia, organism unspecified<br>3. Emphysema	1. Other chronic obstructive pulmonary disease<br>2. Pneumonia, organism unspecified<br>3. Other interstitial pulmonary diseases
	Moderate to profound	1. Pneumonia, organism unspecified<br>2. Pneumonitis due to solids and liquids<br>3. Other chronic obstructive pulmonary disease	1. Other chronic obstructive pulmonary disease<br>2. Pneumonia, organism unspecified<br>3. Other interstitial pulmonary diseases
Diseases of the digestive system	Mild ID	1. Alcoholic liver disease<br>2. Gastric ulcer<br>3-4. Duodenal ulcer; Fibrosis and cirrhosis of liver	1. Alcoholic liver disease<br>2. Fibrosis and cirrhosis of liver<br>3. Acute pancreatitis
	Moderate to profound	1. Paralytic ileus and intestinal obstruction without hernia<br>2. Other diseases of digestive system<br>3. Gastric ulcer	1. Alcoholic liver disease<br>2. Fibrosis and cirrhosis of liver<br>3. Acute pancreatitis
Diseases of the skin and subcutaneous tissue	Moderate to profound	1. Other disorders of skin and subcutaneous tissue, not elsewhere classified<br>2. Ulcer of lower limb, not elsewhere classified<br>3. Cutaneous abscess, furuncle and carbuncle	1. Other disorders of skin and subcutaneous tissue, not elsewhere classified<br>2-4. Cutaneous abscess, furuncle and carbuncle; Other local infections of skin and subcutaneous tissue; Ulcer of lower limb, not elsewhere classified
Diseases of the musculoskeletal system and connective tissue	Mild ID	1. Other systemic involvement of connective tissue<br>2-4. Other rheumatoid arthritis; Scoliosis; Other spondylopathies	1. Other rheumatoid arthritis<br>2. Other necrotizing vasculopathies<br>3-4. Systemic lupus erythematosus;<br>Other systemic involvement of connective tissue
	Moderate to profound	1. Scoliosis<br>2. Other rheumatoid arthritis<br>3-4. Pyogenic arthritis; Coxarthrosis	1. Other necrotizing vasculopathies<br>2. Systemic lupus erythematosus<br>3-4. Other rheumatoid arthritis; Systemic sclerosis
Diseases of the genitourinary system	Mild ID	1. Unspecified kidney failure<br>2. Chronic kidney disease<br>3. Other disorders of urinary system	1-2. Unspecified kidney failure; Other disorders of urinary system<br>3. Chronic kidney disease

© 2021 Hirvikoski T et al. *JAMA Network Open.*

			4 – 10. Chronic nephritic syndrome;<br>Acute tubulo-interstitial nephritis;<br>Tubulo-interstitial nephritis, not specified as acute or chronic;<br>Drug- and heavy-metal-induced tubulo-interstitial and tubular conditions; Calculus of kidney and ureter; Calculus of lower urinary tract;<br>Other disorders of kidney and ureter, not elsewhere classified
	Moderate to profound	1. Other disorders of urinary system<br>2. Unspecified kidney failure<br>3. Calculus of kidney and ureter	1. Unspecified kidney failure<br>2. Chronic kidney disease<br>3. Other disorders of urinary system
**Cause of death category (ICD-10 chapter)**	**ID group (mild ID versus moderate to profound)**	**The three most common causes of death in mild ID**	**The three most common causes of death in reference individuals**
Congenital malformations, deformations and chromosomal abnormalities	Mild ID	1. Spina bifida<br>2-3. Other congenital malformations of heart; Other specified congenital malformation syndromes affecting multiple systems	1. Down syndrome<br>2-4. Congenital malformations of cardiac chambers and connections;<br>Congenital malformations of aortic and mitral valves; Other congenital malformations of heart
	Moderate to profound	1. Down syndrome<br>2. Other congenital malformations of brain<br>3. Microcephaly	1. Down syndrome<br>2-3. Cystic kidney disease;<br>Other specified congenital malformation syndromes affecting multiple systems
Symptoms, signs and abnormal clinical and laboratory findings, not elsewhere classified	Mild ID	1. Other ill-defined and unspecified causes of mortality incl unknown cause of mortality<br>2. Cachexia	1. Other ill-defined and unspecified causes of mortality<br>2. Other general symptoms and signs
	Moderate to profound	1. Other ill-defined and unspecified causes of mortality<br>2-7. Abnormalities of breathing;<br>Other symptoms and signs involving the circulatory and respiratory systems; Dysphagia; Senility;	1. Other ill-defined and unspecified causes of mortality<br>2. Senility<br>3. Other general symptoms and signs

		Haemorrhage, not elsewhere classified; Other general symptoms and signs	
**Cause of death category (ICD-10 chapter)**	**ID group (mild ID versus moderate to profound)**	**The three most common causes of death in mild ID**	**The three most common causes of death in reference individuals**
Injury, poisoning and certain other consequences of external causes	Mild ID	1. Asphyxiation<br>2. Poisoning by narcotics and psychodysleptics [hallucinogens]<br>3. Effects of other external causes	1. Asphyxiation<br>2. Poisoning by narcotics and psychodysleptics [hallucinogens]<br>3. Intracranial injury
	Moderate to profound	1. Foreign body in respiratory tract<br>2. Asphyxiation<br>3. Effects of other external causes	1. Asphyxiation<br>2. Intracranial injury<br>3. Poisoning by narcotics and psychodysleptics [hallucinogens]
External causes of morbidity and mortality	Mild ID	1. Intentional self-harm by hanging, strangulation and suffocation<br>2. Accidental poisoning by and exposure to narcotics and psychodysleptics [hallucinogens], not elsewhere classified<br>3-4. Accidental poisoning by and exposure to other and unspecified drugs, medicaments and biological substances; Exposure to unspecified factor	1. Intentional self-harm by hanging, strangulation and suffocation<br>2. Intentional self-harm by jumping or lying before moving object<br>3. Accidental poisoning by and exposure to narcotics and psychodysleptics [hallucinogens], not elsewhere classified
	Moderate to profound	1. Inhalation and ingestion of food causing obstruction of respiratory tract<br>2. Intentional self-harm by hanging, strangulation and suffocation<br>3. Inhalation and ingestion of other objects causing obstruction of respiratory tract	1. Intentional self-harm by hanging, strangulation and suffocation<br>2. Unspecified fall<br>3. Accidental poisoning by and exposure to narcotics and psychodysleptics [hallucinogens], not elsewhere classified

Note: * ID defined as a cause of death among mild ID 13/1803 (0.72%); among moderate to profound ID 130/5081 (2.56%).

© 2021 Hirvikoski T et al. *JAMA Network Open.*

eTable 6. The Most Common Diagnoses Defined as Potentially Avoidable Deaths (Both Preventable and Amenable), Potentially Preventable Deaths and Deaths Potentially Amenable to Health Care (Definitions Shown in Supplementary Table 3), in ID Cohort 1 Young Adults With Mild ID and the Matched Reference Individuals, as well as ID Cohort 2 All Cases with ID from the National Patient Register and Their Reference Group

Up to ten causes of death are depicted for each category. If the number of cases in the ID group is below 5 in any cell, the data is not shown.

Potentially avoidable death, category	ID group	The most common causes of death in the ID group (% of that category)	The most common causes of death in reference individuals (% of that category)
**Cohort 1. young adults with mild ID born 1980 – 1991**			
Potentially avoidable, all (preventable and amenable combined)	Mild ID	1. Epilepsy (15.74%)<br>2-4. Asphyxiation; Intentional self-harm by hanging, strangulation and suffocation; Poisoning by narcotics and psychodysleptics [hallucinogens] (7.41% each)<br>5. Accidental poisoning by and exposure to narcotics and psychodysleptics (5.56%)	1. Asphyxiation (11.98%)<br>2. Intentional self-harm by hanging, strangulation and suffocation (11.38%)<br>3. Poisoning by narcotics and psychodysleptics [hallucinogens] (9.43%)<br>4-5. Concussion; Accidental poisoning by and exposure to narcotics and psychodysleptics (4.49% each)
Potentially preventable	Mild ID	1-3. Asphyxiation; Intentional self-harm by hanging, strangulation and suffocation; Poisoning by narcotics and psychodysleptics [hallucinogens]; (9.88% each)<br>4. Accidental poisoning by and exposure to narcotics and psychodysleptics (7.41%)	1. Asphyxiation (12.48%)<br>2. Intentional self-harm by hanging, strangulation and suffocation (11.86%)<br>3. Poisoning by narcotics and psychodysleptics [hallucinogens] (9.83%)<br>4. Concussion (4.68%)
Amenable to health care	Mild ID	1. Epilepsy (54.84%)<br>2. Congenital malformations of cardiac septa (9.68%)	1. Acute myocardial infarction (12.50%)<br>2-3. Lymphoid leukaemia; epilepsy (10.00 % each)

© 2021 Hirvikoski T et al. *JAMA Network Open.*

Potentially avoidable death, category	ID group	The most common causes of death in the ID group (% of that category)	The most common causes of death in reference individuals (% of that category)
**Cohort 2. individuals with ID (all severity levels and ages) born 1932-2013**			
Potentially avoidable, all (preventable and amenable combined)	Mild ID	1. Acute myocardial infarction (9.21%)<br>2. Chronic ischaemic heart disease (6.68%)<br>3. Other chronic obstructive pulmonary disease (4.43%)<br>4-5. Epilepsy; Pneumonia (3.88% each)<br>6. Malignant neoplasm of bronchus and lung (2.89%)<br>7. Malignant neoplasm of breast (2.62%)<br>8. Unspecified diabetes mellitus (2.44%)<br>9. Cerebral infarction (2.08%)<br>10. Sequelae of cerebrovascular disease (1.81%)	1. Acute myocardial infarction (9.97%)<br>2. Malignant neoplasm of bronchus and lung (8.73%)<br>3. Chronic ischaemic heart disease (7.37%)<br>4. Malignant neoplasm of breast (3.69%)<br>5. Malignant neoplasm of colon (3.05%)<br>6. Other chronic obstructive pulmonary disease (2.72%)<br>7. Asphyxiation (2.42%)<br>8. Drowning (2.27%)<br>9. Unspecified diabetes mellitus (2.18%)<br>10. Poisoning by narcotics and psychodysleptics [hallucinogens] (2.18%)
Potentially avoidable, all (preventable and amenable combined)	Moderate to profound ID	1. Acute myocardial infarction (10.95%)<br>2. Epilepsy (8.28%)<br>3. Pneumonia (3.88%)<br>4. Chronic ischaemic heart disease (6.44%)<br>5. Stroke (2.46%)<br>6. Malignant neoplasm of colon (2.41%)<br>7. Other sepsis (2.30%)<br>8. Foreign body in respiratory tract (2.30%)<br>9. Other chronic obstructive pulmonary disease (2.25%)	1. Acute myocardial infarction (10.53%)<br>2. Malignant neoplasm of bronchus and lung (8.02%)<br>3. Chronic ischaemic heart disease (6.77%)<br>4. Malignant neoplasm of colon (3.81%)<br>5. Malignant neoplasm of breast (3.40%)<br>6. Asphyxiation (3.02%)<br>7. Other chronic obstructive pulmonary disease (2.88%)<br>8. Drowning (2.84%)<br>9. Concussion (1.88%)

© 2021 Hirvikoski T et al. *JAMA Network Open.*

		10. Unspecified diabetes mellitus (2.10%)	10-11. Aortic aneurysm and dissection; Poisoning by narcotics and psychodysleptics [hallucinogens] (1.87% each)
**Potentially avoidable death, category**	**ID group**	**The most common causes of death in the ID group (% of that category)**	**The most common causes of death in reference individuals (% of that category)**
**Cohort 2. individuals with ID (all severity levels and ages) born 1932-2013**			
Potentially preventable	Mild ID	1. Acute myocardial infarction (12.64%)<br>2. Chronic ischaemic heart disease (9.17%)<br>3. Other chronic obstructive pulmonary disease (6.07%)<br>4. Malignant neoplasm of bronchus and lung (3.97%)<br>5. Malignant neoplasm of breast (3.59%)<br>6. Unspecified diabetes mellitus (3.35%)<br>7-8. Malignant neoplasm of colon; Asphyxiation; Pulmonary embolism; Drowning (2.35% each)<br>9-10. Pulmonary embolism (2.23%); Drowning (2.23%)	1. Acute myocardial infarction (11.69%)<br>2. Malignant neoplasm of bronchus and lung (10.24%)<br>3. Chronic ischaemic heart disease (8.65%).<br>4. Malignant neoplasm of breast (4.30%)<br>5. Malignant neoplasm of colon (3.56%)<br>6. Emphysema (3.17%)<br>7. Asphyxiation (2.82%)<br>8. Drowning (2.64%)<br>9-10. Unspecified diabetes mellitus; Poisoning by narcotics and psychodysleptics [hallucinogens] (2.54% each)
	Moderate to profound ID	1. Acute myocardial infarction (19.74%)<br>2. Chronic ischaemic heart disease (11.61%)<br>3. Malignant neoplasm of colon (4.34%)<br>4. Foreign body in respiratory tract (4.15%)<br>5. Other chronic obstructive pulmonary disease (4.06%)<br>6. Unspecified diabetes mellitus (3.78%)<br>7. Pulmonary embolism (2.93%)	1. Acute myocardial infarction (12.37%)<br>2. Malignant neoplasm of bronchus and lung (9.42%)<br>3. Chronic ischaemic heart disease (7.96%)<br>4. Malignant neoplasm of colon (4.46%)<br>5. Malignant neoplasm of breast (3.98%)<br>6. Asphyxiation (3.54%)<br>7. Other chronic obstructive pulmonary disease (3.38%)

© 2021 Hirvikoski T et al. *JAMA Network Open.*

		8. Malignant neoplasm of breast (2.74%)<br>9. Malignant neoplasm of bronchus and lung (2.55%)<br>10. Inhalation and ingestion of food causing obstruction of respiratory tract (2.17%)	8. Drowning (3.33%)<br>9. Concussion (2.21%)<br>10-11. Aortic aneurysm and dissection; Poisoning by narcotics and psychodysleptics [hallucinogens] (2.19%)
**Potentially avoidable death, category**	**ID group**	**The most common causes of death in the ID group (% of that category)**	**The most common causes of death in reference individuals (% of that category)**
**Cohort 2. individuals with ID (all severity levels and ages) born 1932-2013**			
Amenable to health care	Mild ID	1. Acute myocardial infarction (15.27%)<br>2. Chronic ischaemic heart disease (11.08%)<br>3. Other chronic obstructive pulmonary disease (7.34%)<br>4-5. Epilepsy; Pneumonia (6.44% each)<br>6. Malignant neoplasm of breast (4.34%)<br>7. Unspecified diabetes mellitus (4.04%)<br>8. Cerebral infarction (3.44%)<br>9. Sequelae of cerebrovascular disease (2.99%)<br>10. Malignant neoplasm of colon (2.84%)	1. Acute myocardial infarction (20.57%)<br>2. Chronic ischaemic heart disease (15.21%)<br>3. Malignant neoplasm of breast (7.61%)<br>4. Malignant neoplasm of colon (6.30%)<br>5. Other chronic obstructive pulmonary disease (5.61%)<br>6. Unspecified diabetes mellitus (4.49%)<br>7. Intracerebral haemorrhage (3.74%)<br>8-9. Malignant neoplasm of rectum; Cerebral infarction (2.93%)<br>10. Malignant melanoma of skin (2.56%)
	Moderate to profound ID	1. Acute myocardial infarction (14.63%)<br>2. Epilepsy (11.06%)<br>3. Pneumonia, organism unspecified (9.73%)<br>4. Chronic ischaemic heart disease (8.61%)<br>5. Stroke (3.29%)	1. Acute myocardial infarction (21.42%)<br>2. Chronic ischaemic heart disease (13.78%)<br>3. Malignant neoplasm of colon (7.74%)<br>4. Malignant neoplasm of breast (6.91%)<br>5. Other chronic obstructive pulmonary disease (5.86%)

© 2021 Hirvikoski T et al. *JAMA Network Open.*

		6. Malignant neoplasm of colon (3.22%)<br>7. Other sepsis (3.08%)<br>8. Other chronic obstructive pulmonary disease (3.01%)<br>9. Unspecified diabetes mellitus (2.80%)<br>10. Cerebral infarction (2.73%)	6. Intracerebral haemorrhage (3.51%)<br>7. Unspecified diabetes mellitus (3.36%)<br>8. Malignant neoplasm of rectum (3.00%)<br>9. Cerebral infarction (2.79%)<br>10. Malignant neoplasm of bladder (2.42%)

© 2021 Hirvikoski T et al. *JAMA Network Open.*

eTable 7. Post Hoc Analyses of ID cohort 2, Stratified According to Diagnostic Status of Co-morbid Epilepsy (Yes/No) for Overall and Cause-Specific Mortality, Respectively

In addition to the crude odds ratio (OR with 95% confidence intervals, CI), results from adjusted analyses show OR when adjusted for parental education; congenital malformation and chromosomal abnormalities; epilepsy; autism spectrum disorder and/or ADHD; depression and/or anxiety, respectively. The detailed description of the confounders including diagnostic codes, is provided in Supplementary table 1. The last column depicts ORs adjusted for all above-mentioned potentially confounding factors.

Death cause (ICD-10 Chapter)	ID group and epilepsy diagnostic status	ID n/ N (%)	Reference individuals n/N (%)	OR (95% CI)	Analyses adjusted to potentially moderating factors, OR (95% CI)				Adjusted for all potentially confounding factors OR (95% CI)
				Crude	Parental education	Congenital malformations	ASD, ADHD	Depression, anxiety	
**Overall mortality regardless cause of death**									
All chapters	All, moderate to profound, no epilepsy	2,715/ 16,830 (16.13)	5,865/ 168,300 (3.48)	9.37 (8.80-9.97)	9.29 (8.72-9.90)	8.48 (7.94-9.05)	10.02 (9.39-10.70)	9.11 (8.54-9.71)	8.78 (8.19-9.40)

© 2021 Hirvikoski T et al. *JAMA Network Open.*

All chapters	All, moderate to profound, epilepsy	2,366/ 9,772 (24.21)	2,660/ 97,720 (2.72)	21.56 (19.89-23.37)	21.56 (19.88-23.38)	18.32 (16.78-20.00)	24.44 (22.47-26.60)	21.55 (19.88-23.36)	20.82 (18.98-22.83)
All chapters	Males, Moderate to profound, no epilepsy	1,572/ 9,878 (15.91)	3,798/ 98,780 (3.84)	8.34 (7.68-9.05)	8.25 (7.60-8.96)	7.61 (6.99-8.28)	9.11 (8.37-9.93)	8.01 (7.38-8.70)	8.05 (7.36-8.80)
All chapters	Males, Moderate to profound, epilepsy	1,331/ 5,461 (24.37)	1,786/ 54,610 (3.27)	17.69 (15.97-19.59)	17.65 (15.93-19.56)	14.89 (13.33-16.64)	20.39 (18.32-22.70)	17.66 (15.94-19.56)	17.41 (15.48-19.58)
All chapters	Females, Moderate to profound, no epilepsy	1,143/ 6,952 (16.44)	2,067/ 69,520 (2.97)	11.07 (10.03-12.23)	11.02 (9.98-12.18)	9.93 (8.95-11.01)	11.45 (10.34-12.67)	10.94 (9.89-12.09)	9.92 (8.92-11.04)
All chapters	Females, Moderate to profound,	1,035/ 4,311 (24.01)	874/ 43,110 (2.03)	29.03 (25.42-33.15)	29.15 (25.51-33.30)	25.23 (21.83-29.15)	32.05 (27.92-36.79)	29.03 (25.42-33.15)	27.32 (23.50-31.76)

© 2021 Hirvikoski T et al. *JAMA Network Open.*

	epilepsy								
**Specific cause of death**					**Analyses adjusted to potentially moderating factors OR (95% CI)**				**Adjusted for all potentially confounding factors OR (95% CI)**
**Death cause (ICD-10 Chapter)**	**ID group and epilepsy diagnostic status**	**ID n/ N (%)**	**Reference individuals n/N (%)**	**Crude OR (95% CI)**	**Parental education**	**Congenital malformations**	**ASD, ADHD**	**Depression, anxiety**	
VI Nervous system	Mild, no epilepsy	55/ 20,250 (0.27)	134/ 202,500 (0.07)	4.13 (3.01-5.65)	4.13 (3.01-5.67)	3.82 (2.77-5.27)	4.12 (2.99-5.69)	4.16 (2.98-5.82)	3.75 (2.67-5.29)
VI Nervous system	Mild, epilepsy	87/ 3,809 (2.28)	18/ 38,090 (0.05)	48.33 (29.10-80.29)	49.61 (29.78-82.62)	48.83 (29.18-81.72)	47.62 (28.30-80.12)	52.92 (31.69-88.39)	52.84 (30.79-90.69)
VI Nervous system	Moderate to profound, no epilepsy	236/ 16,830 (1.40)	178/ 168,300 (0.11)	13.52 (11.11-16.46)	13.61 (11.16-16.60)	12.52 (10.19-15.39)	14.57 (11.91-17.82)	14.15 (11.60-17.25)	13.63 (11.00-16.89)

© 2021 Hirvikoski T et al. *JAMA Network Open.*

VI Nervous system	Moderate to profound, epilepsy	642/ 9,772 (6.57)	99/ 97,720 (0.10)	72.31 (57.86-90.36)	72.64 (58.10-90.81)	72.29 (57.27-91.24)	81.93 (65.28-102.81)	72.67 (58.15-90.81)	82.48 (64.68-105.17)
**Specific cause of death**					**Analyses adjusted to potentially moderating factors OR (95% CI)**				**Adjusted for all potentially confounding factors OR (95% CI)**
**Death cause (ICD-10 Chapter)**	**ID group and epilepsy diagnostic status**	**ID n/ N (%)**	**Reference individuals n/N (%)**	**Crude OR (95% CI)**	**Parental education**	**Congenital malformations**	**ASD, ADHD**	**Depression, anxiety**	
XVII Mal-formations, chromosomal	Moderate to profound, no epilepsy	251/ 16,830 (1.49)	29/ 168,300 (0.02)	86.55 (58.93-127.12)	91.33 (62.06-134.41)	29.52 (19.70-44.22)	103.57 (70.02-153.21)	94.05 (63.97-138.27)	37.00 (24.49-55.92)
XVII Mal-formations, chromosomal	Moderate to profound, epilepsy	297/ 9,772 (3.04)	17/ 97,720 (0.02)	185.28 (112.04-306.40)	186.71 (112.86-308.89)	58.45 (34.29-99.62)	236.42 (142.19-393.08)	186.41 (112.72-308.28)	66.99 (39.16-114.61)

Note: %: Percent; OR: odds ratio; CI: confidence interval.

© 2021 Hirvikoski T et al. *JAMA Network Open.*

eFigure 1. Flowchart of the Two Included Cohorts

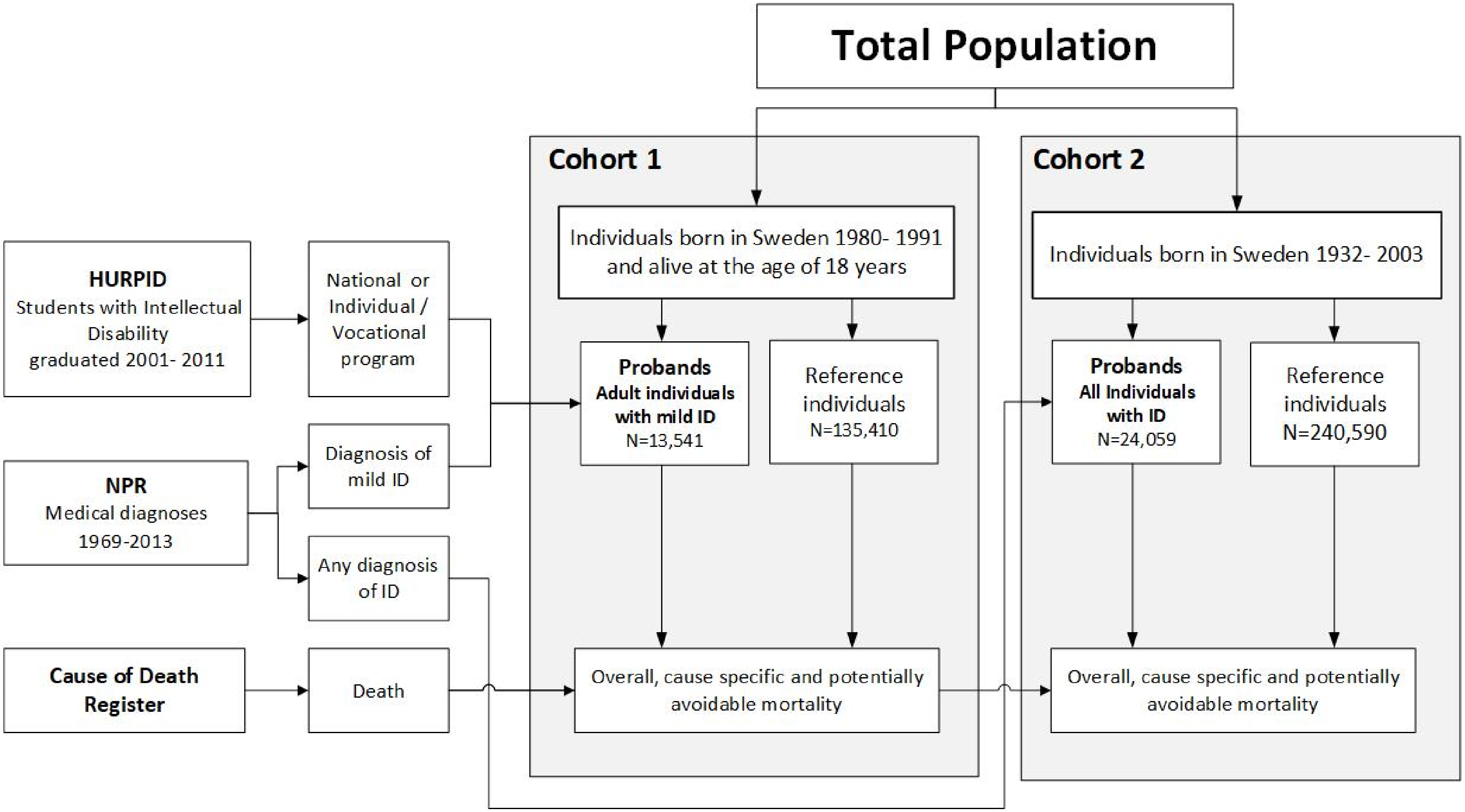

© 2021 Hirvikoski T et al. *JAMA Network Open.*

eFigure 2. Kaplan-Meier Survival Estimators

Young adults with mild ID (born 1980-1991), and matched reference individuals are shown in upper left panel, and close-up of the same groups in upper right panel. ID cohort 2 (all ages, born 1932-2013) is shown grouped into mild ID and their reference individuals (lower left panel) and moderate to profound ID and their reference individuals (lower right panel).

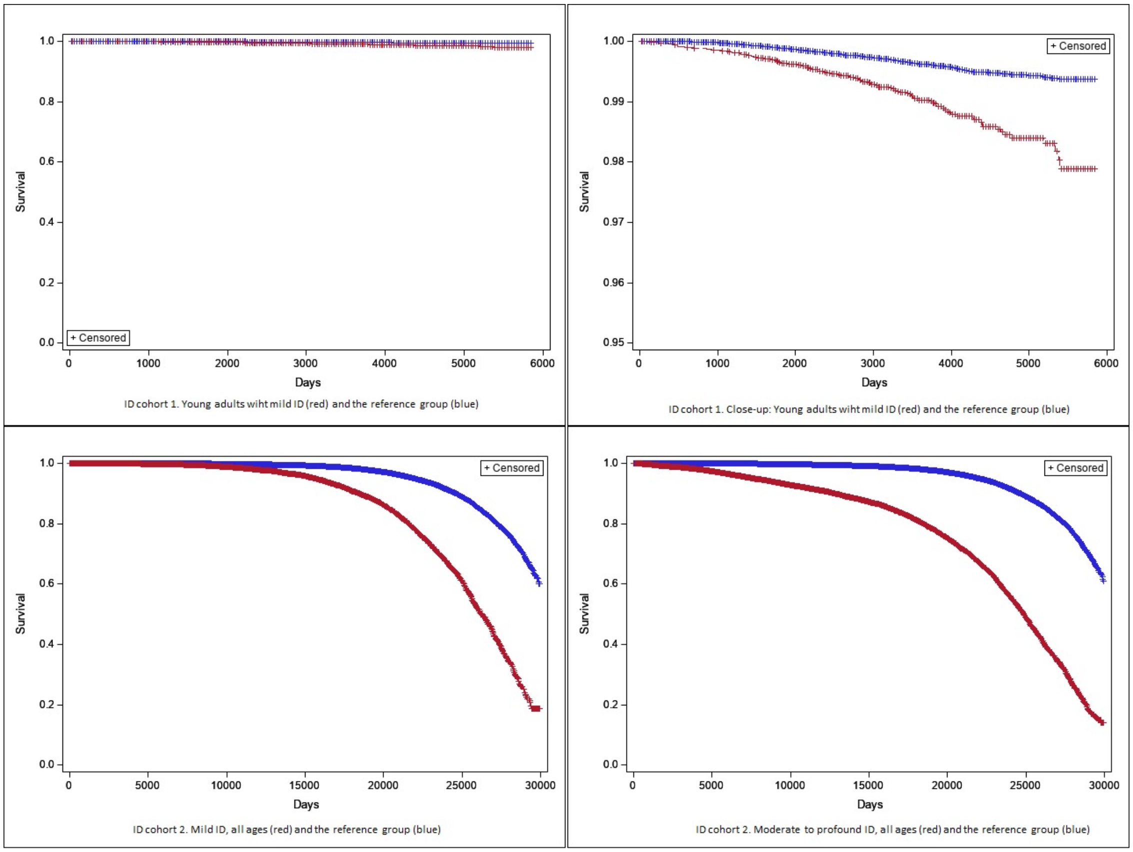

© 2021 Hirvikoski T et al. *JAMA Network Open.*
